# Canada's Patented Medicine Notice of Compliance regulations: balancing the scales or tipping them?

**DOI:** 10.1186/1472-6963-11-64

**Published:** 2011-03-24

**Authors:** Joel Lexchin

**Affiliations:** 1 School of Health Policy and Management, York University, 4700 Keele St., Toronto Ontario M3J 1P3, Canada; 2 Department of Emergency Medicine, University Health Network, 190 Elizabeth St. Toronto Ontario M5G 2C4, Canada; 3 Department of Family and Community Medicine, University of Toronto, 163 McCaul St. Toronto Ontario M5G 1L5, Canada

## Abstract

**Background:**

In order to comply with the provisions of the North American Free Trade Agreement, in 1993 the Canadian federal government introduced the Patented Medicine Notice of Compliance Linkage Regulations. These regulations were meant to achieve a balance between the timely entry of generic medicines and the rights of patent holders. The regulations tied the regulatory approval of generic medicines to the patent status of the original brand-name product.

**Discussion:**

Since their introduction the regulations have been a source of contention between the generic and the brand-name industry. While the regulations have generated a considerable amount of work for the Federal Court of Canada both sides dispute the interpretation of the "win rate" in the court cases. Similarly, there is no agreement on whether multiple patents on single drugs represent a legitimate activity by the brand-name industry or an "evergreening" tactic. The generic industry's position is that the regulations are being abused leading to the delay in the introduction of lower cost generic products by as much as 8 years. The brand-name companies counter that the regulations are necessary because injunctions against the introduction of generic products are frequently unavailable to them. The regulations were amended in 2006 and again in 2008 but both sides continue to claim that the regulations favour the other party. The battle around the regulations also has an international dimension with interventions by PhRMA, the trade association representing the United States based multinational companies, arguing that the regulations are not stringent enough and that Canada needs to be placed on the U.S. Priority Watch List of countries. Finally, there are multiple costs to Canadian society as a result of the NOC regulations.

**Summary:**

Despite the rhetoric there has been almost no empiric academic research done into the effect of the regulations. In order to develop rational policy in this area a number of key research questions have been formulated.

## Background

Countries are increasingly reliant on generic medications to keep drug costs under control. In Canada although more than 50% of all prescriptions are written generically, they account for only 24% of total spending[1]. The use of generics puts governments in a contradictory role - on the one hand they need generics to keep spending under control but on the other hand they need to restrict the entry of generics to encourage brand-name company investment. One key manifestation of this contradiction is in the Patented Medicine Notice of Compliance (NOC) linkage regulations that tie the regulatory approval of generic medications by Health Canada to the patent status of the original brand-name product.

This paper starts by reviewing the origins and operations of these linkage regulations. It then looks at two contentious issues - the outcome of judicial hearings and the consequences of multiple patents on individual drugs. Linkage regulations are one of the battlegrounds between the generic and brand-name arms of the industry and the positions of the organizations representing these companies are examined. In 2006 and 2008 the regulations were amended and the next section looks at the reasons why changes were made and their potential impact. Linkage regulations are not just a domestic concern, the Pharmaceutical Research and Manufacturers of America (PhRMA), the body representing brand-name companies in the United States, has been an active participant in the debate about them and the following section addresses its arguments. The penultimate section discusses how the linkage regulations are not just a matter of a dispute between brand and generic companies. There are multiple costs to Canadian society as a result of their existence. Finally, the paper concludes by posing a series of questions that future research should examine.

### Origin of linkage regulations

In 1993, in order to comply with the requirements of the North American Free Trade Agreement (NAFTA) Canada abolished its system of using compulsory licensing of pharmaceuticals as a cost control tool and established a 20-year patent term for medications[2]. In addition, the government introduced the NOC linkage regulations, meaning that Health Canada, the national drug regulatory agency, was prevented from issuing an authorization for market entry for a generic until all of the relevant patents on the brand-name product had been proven to have expired.

The government's explanation for the linkage regulations were that they were part of its pharmaceutical patent policy that sought "to balance effective patent enforcement over new and innovative drugs with the timely market entry of their lower priced generic competitors."[3] This explanation for the necessity to introduce these regulations was echoed by the brand-name industry[4]. One part of the balance was allowing generic companies to begin testing their products before the patent had expired on the original product in order for the generic product to be in a position to enter the market as soon as possible after patent expiry. Having removed a right available to all other patentees the linkage regulations were introduced "...to ensure that this new exception to patent infringement [was] not abused by generic drug applicants seeking to sell their products during the term of the competitor's patent."[3]

As a result, when the generic company submits its application to get a product approved it also sends a Notice of Allegation (NOA) to the patent holder claiming that no patents are being infringed. The patent holder then has 45 days in which to initiate an application in the Federal Court of Canada seeking an order to prohibit Health Canada from issuing a NOC to the generic manufacturer for a period of 24 (originally 30) months. At that point, the matter may proceed to a court hearing. The stay expires either at the end of the 24 months, when the patent expires or when the court case is decided, whichever comes first[5].

### Administration of linkage regulations

The NOC linkage regulations were formulated by Industry Canada and are administered by the Office of Patented Medicines and Liaison (OPML), located in the Therapeutic Products Directorate, Health Products and Food Branch, Health Canada. They require the Minister of Health to maintain a Patent Register (http://www.patentregister.ca/). This consists of patent lists submitted in respect of eligible NOC-issued drugs, i.e., drugs that have been approved for marketing[5].

The Minister responsible for Health Canada may refuse to add, or may delete, information from this Patent Register. Each patent list is audited (for example as to whether potential inclusions are mere "evergreening" attempts, i.e., attempts to unfairly extend patent life in order to prevent generic competition) by the OPML. Reports produced by that body provide statistics relating to the maintenance of the Patent Register, including the number of patents filed, the number of patents accepted and rejected, and litigation resulting from the acceptance or rejection of patents for listing on the Patent Register[6]. According to the 2008 report, from 1998 to 2008 there were an average of 54 NOAs received per year and 41 court applications were commenced per annum[6].

## Discussion

### Judicial hearings

The Canadian Generic Pharmaceutical Association (CGPA) claims that between 1998 and 2003, the generic companies won 80% of the court cases[7]. Canada's Research-Based Pharmaceutical Companies (Rx&D) disputes the 'win rate' for the generic companies in court cases and presents its own figures showing that generic and patentee 'wins' about balance each other out. However, Rx&D is interpreting the data in a potentially biased manner. At the time when Rx&D presented its statistics, there were 125 cases where there was no hearing; the 20 cases where the NOA was withdrawn were counted as a win for the patentee but the 100 cases where the innovator either accepted the NOA or the case was otherwise settled were not counted as wins for the generic[8].

Overall between 1998 and 2008, the latest year for which statistics are available, there have been 447 cases where a court application was commenced. In 97 cases the prohibition against issuing the generic company a NOC was dismissed, prohibitions were granted 53 times (in 5 cases there was a partial prohibition, i.e., more than one patent was being disputed but the prohibition did not apply to all patents) and there are 78 prohibitions pending the resolution of the court case. (See Table 1.) Out of 357 cases that have been closed, 13% (46) have taken more than 24 months to resolve[6]. Although the federal government intended the NOC proceedings to be summary in nature and therefore of short duration, in practice this has not proved to be the case[9]. The linkage regulations have also created a significant backlog in the Canadian Federal Court system. In 2008, there was a team of approximately 30 Federal Court judges devoting some or all of their time to about 350 separate drug patent cases[10]. Furthermore, "The Supreme Court of Canada has, on multiple occasions, held that the automatic stay issued to patentees under the *NOC Regulations *is an 'extraordinary' remedy, not available to patentees in any industry outside of the pharmaceutical industry."[9]

**Table 1:** Outcome of court cases, 1998 - 2008

Category	Number
Court applications commenced	447

Partial prohibitions granted	5

Prohibitions pending resolution	78

Prohibitions discontinued	219

Prohibitions granted	48

Prohibitions dismissed	97

Source: Office of Patented Medicines and Liaison [6]

### Multiple patents on single drugs

A particularly contentious tactic is the use of multiple patents by brand-name companies to delay the appearance of a generic product. The CGPA's contention is that the brand-name companies continually listed new patents on a product, each of which can trigger a new NOA and an additional stay on the appearance of a generic. In this way, competition is delayed[7]. The use of multiple patents involves about half the products with registered patents. As of the end of December 2008 there were 494 medicines listed on the Patent Register. Of these 248 had one patented registered, 120 had two and out of the remaining 126, 40 had more than 5 patents (one of these had 22 patents)[6]. In the past, these multiple patents, filed over a period of time, had the effect of delaying a final decision about marketing a generic product for 8 years[11].

The brand-name companies maintain that multiple patents are not an issue. Their position is that there is always ongoing research into drugs and that it is natural that new patents will be filed, reflecting improvements such as moving from a three pill a day regimen to once a day dosing. The multinationals also say that in 95% of cases all subsequent patents will be issued within 10 years of the initial patent and therefore all of the patents may be addressed in the same linkage proceeding. But if the effective patent life is only 10 years, as Rx&D claims it is, then new patents are being filed as old ones expire. Even if patent life is a couple of years beyond 10 years there can still be overlapping 24-month stays depending on when the generic company files for a NOC. According to Rx&D in that situation, and in the situation where court cases take more than 24 months to resolve, all the generic companies have to do is market the older version of the product on expiry of the original patent[8]. However, this argument ignores the fact that the main reason for launching a new formulation of a drug is to switch doctors to that version before a generic is available in order to undercut the market for the generic. The marketing of new formulations is something that brand-name companies spend millions of advertising dollars doing.

Finally, the acknowledgement that there are multiple patents on single products, even though there is disagreement regarding their effects, is ironic. During the debate leading up to the 1993 legislation establishing the linkage regulations the Minister of Industry, Mining and Technology and the Director General of the Chemical and Bio-Industries Branch in the Department of Industry, Science and Technology both strenuously maintained that each drug had a single main patent and once that patent expired anyone could copy the drug and bring it to market[12].

### Canadian Generic Pharmaceutical Association

The Canadian Generic Pharmaceutical Association (CGPA) claims that "not only is this abuse [i.e., the linkage regulations] of Canada's patent regime extremely harmful to Canada's generic pharmaceutical industry, the Canadian public loses out on millions of dollars in savings by having to pay for the higher-priced brand-name version for an extended period of time. The delays caused by these needless court battles have cost Canadians, their governments and private insurers hundreds of millions of dollars."[7]

The CGPA also contends that the regulations place generic companies in the position of facing "double jeopardy". Even if a generic company is successful in litigation under the linkage regulations and gets its product on the market, it can still be sued again on the same patents it has just litigated. This situation exists because litigation under the regulations does not result in a final determination by the court regarding whether the patent in question is valid or infringed[11]. Since the NOC system is not designed to provide for a full exploration of the validity of a patent, for some products there are multiple court proceedings. According to a recent report commissioned by the CPGA the possibility that the patentee may subsequently sue for infringement is a barrier to the entry of generic drugs[9].

### Canada's Research-Based Pharmaceutical Companies

Canada's Research-Based Pharmaceutical Companies (Rx&D) counters that these regulations are necessary because "generics do not have to concern themselves with a possible interlocutory injunction [a temporary injunction that lasts only until the end of the trial during which the injunction was sought] to prevent infringing sales once an infringing generic product is on the market. Statistics show that this remedy is available in pharmaceutical cases approximately half as often as in other industry patent cases. Indeed, as a result of the inability of pharmaceutical patentees to obtain interlocutory injunctions to prevent the complete destruction of their intellectual property rights and market share, the linkage regulations are the only means for Canada to meet its international obligations to provide an effective enforcement mechanism for patents."[8] In-other-words, Rx&D's claim is that the availability of injunctions against the marketing of a generic product, the remedy that would be available in the absence of the linkage regulations, is insufficient protection because these injunctions are frequently unavailable to the brand-name companies.

### 2006 and 2008 NOC amendments to the regulations

Generic companies had long been complaining that brand-name companies were abusing the linkage regulations by adding on irrelevant new patents,[11] but criticisms about the regulations were also coming from other sources. The Competition Bureau rejected a complaint from the National Union of Public and General Employees that brand-name companies were using the claim of patent infringement as a way to delay the entry of generic drugs. However, at the same time the Bureau noted that "A number of court decisions over the last several years regarding what constitutes a relevant patent and the time period during which such a patent can be added have somewhat altered the balance contained in the NOC Regulations between the competing interests of the brand name pharmaceutical patent holders and generic drug companies. There is also no ready mechanism in the NOC Regulations for compensating consumers affected by delays in the introduction of generic drugs, thereby creating a possible incentive for brand name pharmaceutical companies to strategically use the NOC Regulations to improperly delay generic drug entry."[13]

In October 2006, the Canadian federal government seemingly recognized the validity of these criticisms and limited brand-name companies use of follow-on patents by amending the regulations. These amendments prevented any new patents that the brand-name companies filed after a generic company had submitted an application for approval of its product from being considered in the linkage regulations process. Moreover the new regulations made it clear that patents covering areas without direct therapeutic application, such as processes or metabolic intermediates, could not be used to delay generic approval[3]. Less than two months later the Supreme Court of Canada also recognized that the brand-name companies had been abusing the linkage regulations by adding irrelevant patents[14].

Although the change described above was favourable to the interests of the generic companies, there were also changes that they viewed less favourably. To discourage abuse of the linkage regulations by the brand-name companies, section 8 of the regulations had previously allowed generic companies to collect "damages or profits" from the brand-name company when the generic drug was wrongfully kept off the market by the automatic 24 month stay. One of the 2006 amendments removed the words "or profits" from section 8 meaning that brand-name companies could no longer be penalized with the loss of their profits acquired during the period in which a court found that the brand has abused the automatic stay provision. The position of the CGPA was that "The Government of Canada has essentially made it less economic for anyone to make the investment necessary to challenge the brand-name company's monopoly, and introduce a low-cost generic product to market."[15]

Regulations were further amended in 2008. The government argued that these new amendments were necessary because of a Federal Court of Canada ruling that changed the intent of the 2006 amendments. The intent of the these amendments was to exempt ("grandfather") patents submitted for listing that were in conformity with the pre-2006 rules from coming under the 2006 changes. The Federal Court ruling was seen as undermining that protection and as a consequence there was a concern that many patents submitted in full compliance with the listing requirements, as they were interpreted and applied prior to June 17, 2006, could be deleted from, or not added to, the Patent Register. This could result in earlier than anticipated loss of market exclusivity for a number of brand-name drugs. The new regulations prohibited the deletion of grandfathered patents from the Patent Register and prohibited the government from refusing to add any patent solely on the basis of the Federal Court ruling[16].

When it introduced the 2008 amendments, the federal government acknowledged that they "'could result in delayed savings to consumers and provincial drug plans.' But those concerns were counterbalanced, it said, by the pharmaceutical industry's need to have confidence in Canada as a place to invest in research and development." Whether or not companies continued to view Canada as a good place to invest despite the amendments is debatable. In July 2010 Merck Frosst announced its decision to close its Montreal research facility and lay off most of the 200 employees[17].

### Pharmaceutical Research and Manufacturers of America (PhRMA)

PhRMA regards the 2008 regulation changes as a positive step but at the same time maintains that "serious and systematic deficiencies remain with the PM [Patented Medicines] NOC Regulations."[18] PhRMA bases its position on three points: brand-name companies have no effective right of appeal; there are limitations on the listing of valid patents; patent infringement proceedings need strengthening.

On the first point, PhRMA argues that if the generic company wins the court case under the summary proceeding, aimed only at determining if the allegation is justified, and is allowed to market its product, then once a NOC has been issued any appeal filed by the patentee becomes moot. As a result of the summary nature of the proceeding, there is no discovery and there may be constraints on obtaining and introducing evidence and cross-examination. "The patentee is then left with no alternative but to start another proceeding (an action for patent infringement) once the generic enters the market." In contrast, according to PhRMA, the right of appeal is still available to a generic producer if it loses its initial court case under the summary proceeding[18].

On the second point, PhRMA claims "Patent owners are prevented from listing their patents in the Patent Register established under the PM (NOC) Regulations if the patents do not meet certain arbitrary timing requirements or are of a type not eligible for listing." Finally, PhRMA says that while brand-name companies may choose to try and protect their patent rights through an interlocutory injunction "to prevent the market entry of the generic product or to seek its withdrawal from the market, these motions rarely succeed in Canada even if there is compelling evidence of infringement. Additionally, it takes years before an action for patent infringement is tried. By then the innovative company's market share can be severely eroded by the marketing of the generic product."[18]

In light of these (and other) problems with respect to the way that Canada deals with prescription medicines PhRMA "requested that Canada remain on the **Priority Watch List **for the 2010 Special 301 Report and that the U.S. Government continue to seek assurances that the problems described herein are quickly and effectively resolved (Emphasis in original)." The Special 301 Report is an annual publication issued by the United States Trade Representative which examines in detail the adequacy and effectiveness of intellectual property rights in countries around the world. Based on this report countries may be designated in the categories of Priority Watch List, Watch List, and/or Section 306 Monitoring status. Although Canada was put on the Priority Watch List in 2009 and remained on it in 2010 the main reason was its lack of action on the question of copyright reform. Patent protection for pharmaceuticals was not mentioned in the report[19].

### Cost to Canadian society

The linkage regulations are not just a matter of a dispute between brand-name and generic companies. There are multiple costs to Canadian society associated with their existence starting with the administrative costs of maintaining the Patent Register to the costs to the judicial system from the multiple patent litigation cases. In addition, there are costs to the provincial drug plans. The increase in drug spending attendant on the delay in the appearance of generic equivalents may partly explain why provincial governments have increased co-payments and deductibles for recipients of publicly funded drug plans. The CGPA claims that the 2008 amendments will cost consumers and taxpayers tens of millions of dollars annually[20]. The projected increase in costs from these amendments prompted the governments in British Columbia and New Brunswick to oppose them[21]. New Brunswick estimated that it would add $4 million to its annual drug bill of $162 million[22]. Finally, the NOC regulations allow generic companies to seek lost profits if they are successful in NOC proceedings. However, there is no compensation to payers (public, private or those who paid out of pocket) who paid brand prices instead of generic ones[9].

## Summary

The putative reason for the NOC linkage regulations was to provide a balance between the economic interests of the brand-name and generic pharmaceutical companies. While these regulations have certainly provided work for the court system, despite having been on the books for over 17 years there has been almost no empiric research undertaken into their effects. At present, most of the discussion about the regulations consists of claims and counterclaims from supporters of one side of the debate or the other. Among the unanswered questions are the following:

1. How might the position of PhRMA regarding the Canadian regulations affect overall trade relations between Canada and the United States?

2. Do the regulations affect investment decisions by either generic or brand-name companies?

3. Do the regulations discourage generic companies from introducing some drugs?

4. Do the regulations affect the value of drugs exported from or imported into Canada?

5. What is the sum of the added costs incurred by the provinces as a result of the regulations because of the delay in the introduction of generics and because during that delay brand-name companies have convinced doctors to switch to a patent protected version of the product or a new product?

6. Do the regulations lead to R&D investment into ways of modifying products with a view to "evergreening" as opposed to investment in innovative products?

7. What are the legal costs associated with the regulations from the point of view of all parties - federal and provincial governments and the generic and brand-name companies?

Answers to questions such as these will help guide the government in future policy decisions about the regulations and allow a determination of whether or not the regulations are actually balancing the scales or tipping them in favour of one side.

## Authors' contributions

JL conceived this paper, gathered the references and is solely responsible for writing the manuscript.

## Pre-publication history

The pre-publication history for this paper can be accessed here:

http://www.biomedcentral.com/1472-6963/11/64/prepub
